# Performance of Jazia Prime Vendor System in ensuring availability of health commodities in Singida Region, Tanzania: a pre- and post-evaluation study

**DOI:** 10.1186/s40545-023-00660-y

**Published:** 2023-11-20

**Authors:** Daniel Pyuza, Shiferaw Mitiku, Omary Swalehe, Vedaste Kagisha, Kato Jonas Njunwa

**Affiliations:** 1 EAC Regional Centre of Excellence for Vaccines, Immunization, and Health Supply Chain Management, College of Medicine and Health Sciences, University of Rwanda, P.O. Box 3286, Kigali, Rwanda; 2 Ministry of Health, P.O. Box 743, Dodoma, Tanzania; 3 Addis Ababa University, P.O Box 31520, Addis Ababa, Ethiopia; 4 Mzumbe University, P.O Box 1, Morogoro, Tanzania; 5 School of Pharmacy, College of Medicine and Health Sciences, University of Rwanda, P.O. Box 3286, Kigali, Rwanda; 6 College of Medicine and Health Sciences, University of Rwanda, P.O. Box 3286, Kigali, Rwanda

**Keywords:** Jazia Prime Vendor System, Availability of the health commodities, Singida Region

## Abstract

**Background:**

Availability of the health commodities in public health facilities in Tanzania remains a challenge, and has been reported to be below 70%. Moreover, Medical Stores Department’s capacity to supply health commodities has been only 40%. Therefore, Jazia Prime Vendor System (Jazia PVS) was outsourced to complement the Medical Stores Department. In 2017 Jazia PVS was introduced in Singida Region after being piloted in three other Regions. This study therefore, was conducted to assess the performance of Jazia PVS in enhancing the availability of the health commodities in the public health facilities between 2017 and 2019 in Singida Region, Tanzania.

**Methods:**

A mixed method pre- and post-evaluation analytical study design was used in all the selected public health facilities in the Municipal and District Councils of Singida Region, Tanzania. These included 138 public health facilities: One Regional Referral Hospital, four District Hospitals, 19 Health Centres and 114 Dispensaries. Percent availability of health commodities was abstracted from electronic logistics management information system. Documentary review involved quarterly orders, Jazia PVS delivery notes, and payment vouchers; while all the 138 pharmacists incharge were interviewed.

**Results:**

The mean availability of health commodities was modestly higher after adoption of Jazia PVS (mean = 59.17%, SD = 6.12%) than before Jazia PVS (mean = 54.39%, SD = 5.36%); and the difference between means was 4.78% (*t* = -9.49, *df* = 136, *p* < 0.001). Furthermore, 20.3% (109/421) of orders were fulfilled, while 58% (312/421) were not, (*χ*^2^ = 10.46, *df* = 6, *p* = 0.1067). About 73.7% of orders (320/434) were delivered on time, while 26.3% (114/434) delayed, (*χ*^2^ = 121, *df* = 6, *p* < 0,001). Prompt payment to Jazia PVS was 43.0% (164/381) deliveries, while 57.0% (217/382) were not punctual, (*χ*^2^ = 26, *df* = 6, *p* < 0.001). Satisfaction level of the pharmacists incharge for Jazia PVS was 11.8%, (*χ*^2^ = 78.04, *df* = 3, *p* < 0.001).

**Conclusion:**

With Jazia PVS, availability of health commodities improved by 4.78% in 2 years. Prompt payment of Jazia PVS will enhance performance of the vendor.

## Background

Continuous and adequate availability of health commodities in health facilities is key towards attaining universal health coverage by 2030 [1, 2]. However, worldwide, it is estimated that 30% of the population have no regular access to essential medicines; while in low income countries, most of which are in sub-Saharan Africa, over 50% of the population lack regular provision of essential health commodities including diagnostics, affordable, safe, and effective medicines [3, 4]. Availability of health commodities in sub-Saharan Africa is faced with several challenges along the supply chain system, including procurement performance, logistical, infrastructural, and financial matters, resulting in frequent stock-outs [5].

In Tanzania, the Medical Stores Department (MSD) is the only government agency mandated to procure, store, and distribute health commodities to all public health facilities, from primary to national level countrywide [6]. The central government disburses funds directly to individual health facility accounts in MSD; and by debiting their accounts based on their orders, MSD delivers health commodities to health facilities [6]. MSD has 10 operational zones, of which some encompass more than one Region. MSD zonal offices are based in Dar-es-Salaam, Dodoma, Iringa, Kagera, Kilimanjaro, Mbeya, Mwanza, Mtwara Tabora, and Tanga, [7]. Singida and Dodoma belong to the zone of Dodoma. In the effort to improve the supply chain of medical commodities in Tanzania, MSD underwent considerable reforms in 2015–2016 related to management, operations and revenue generation [8]. The MSD also redesigned its logistic system, to enhance performance, and acquired large and suitable storage as well as transport facilities. By 2018–2019 availability of health commodities had increase by 12.6% compared to the 2015–2016 pre-intervention period [8].

Despite the achievement attained in increased availability of health commodities and the spreading of the electronic logistics management information system (eLMIS) countrywide, in 2015/2016, the order fill rate, which is the percentage of order fulfilled, from zone to the facility gradually fell from 64.2 to 55.4% in 2017/2018 [8, 9]. Likewise, over the corresponding period order fill rate from Central stores to zones fell from 73.4 to 54.0% [8]. This situation translated into continued shortages of health commodities at health facilities. Studies on supply chain performance of MSD showed that until 2020 MSD was able to fulfil about 40% of the facility’s requirements [9]. This low MSD performance arising from poor fulfilment of orders is considered to be the cause of frequent stock-outs at health facilities, [9]. The 2020 reports from the Ministry of Health (MOH) showed that the availability of the essential items countrywide was about 70%, which did not conform to the 80% threshold recommended by WHO [10]; besides, availability of health commodities tends to decline towards the peripheral health facilities (dispensaries and health centres) [11]. Increasing orders from health facilities to MSD for health commodities is considered to be one of the reasons for the low MSD performance and regular stock-outs of health facilities [12, 13].

To strengthen public supply chain management with respect to availability of health commodities, strategic outsourcing system (SOS) approach has been adopted in a number of countries [14]. In this regard, Tanzania, introduced SOS known as Jazia Prime Vendor System (Jazia PVS) in November 2018 [15]. Jazia PVS complements regular government supply by MSD through a Regional contract approach. It operates on public private partnership model, through a Regional pooled agreement approach [16], by which the District or Municipal Councils in each Region make up for the MSD deficit in the supply of medicines and other health commodities. Furthermore, Jazia PVS has been incorporated in the local government structure for increased ownership [16]. Payment for orders of health commodities supplied by Jazia PVS is made by the health facilities using direct health facility funds (DHFF) from the central government to the health facility bank account [12]. The DHFF comprised health basket fund (from development partners) and health insurance funds; some health facilities do raise some additional funds by charging out-of-pocket user fees [11].

However, contrary to what was expected, health facilities have continued to have shortages of health commodities, resulting in patients being obliged to seek for more costly medicines in outlets outside the premises of the health facilities [14]. Despite this situation, available published reports of Jazia PVS performance cover only three Regions namely Dar-es-Salaam, Dodoma and Arusha out of the 21 served by the scheme [12, 15].

Hence, the purpose of this study was to assess the performance of Jazia PVS in complementing the MSD to deliver health commodities in order to improve their availability in Singida Region, Tanzania. The study was conducted in 2017 (before) and 2019 (after) the introduction of Jazia PVS. Performance of Jazia PVS was assessed using the following indicators expressed as percentages: availability of health commodities, order fill rate, and order lead time. Extent of timely payment by health facilities to Jazia PVS, and the level of satisfaction of the pharmacists incharge were also assessed.

## Methods

### Study design and setting

This mixed method pre and post-evaluation analytical study was conducted in public health facilities in Singida Region, Tanzania, during the period 2017 to 2019. Singida Region neighbours Dodoma Region in which Dodoma, the capital city of Tanzania, is located. At the time of the study, Singida Region consisted of 209 public health facilities, namely one Regional Referral Hospital, four District Hospitals, 19 Health Centres, and 185 dispensaries. These health facilities are distributed in seven administrative Councils: Singida Municipal Council, and the District Councils of Ikungi, Iramba, Manyoni, Mkalama, Singida and Itigi.

### Study population and eligibility criteria

The study population consisted of 209 public health facilities, all of which were eligible for the study, since they were the only ones served by Jazia PVS which complements MSD in the supply of health commodities. Also eligible, were the signed and recorded delivery notes, payment vouchers related to health commodities, and all the pharmacists incharge of the pharmacy department of public health facilities in Singida Region.

### Sample size and sampling procedures

The sample size included all of the following public health facilities located in Singida Region: 19 Health Centres, four District Hospitals, and one Regional Referral Hospital. In addition, a sample size of 114 out of 186 dispensaries in all the District Councils was obtained using Yamane formula [17]; and the overall sample size of the health facilities was 138. All the District Hospitals and all Health Centres were selected because they were fewer, and due to the higher number of items they ordered through Jazia PVS. The number of dispensaries selected from each District Council was determined proportionately based on the number of dispensaries in each Council. In each District Council, dispensaries to be used in the study were identified by convenient sampling for logistical reasons. At health facility level, all the 138 pharmacists incharge, all the health facility quarterly orders, Jazia PVS delivery notes, and payment vouchers related to health commodities were included in the study.

### Data collection instruments, procedures, and quality control

Information from the eLMIS about health commodity percentage availability was abstracted using a form developed by the researcher. It included data for 2017 before Jazia PVS started its operation, and data for 2019 after the implementation of Jazia PVS activities. Checklists were used to record paper-based orders, Jazia PVS delivery notes, and payment vouchers from the pharmacy department. Data on order fill rate, of Jazia PVS to health facilities were calculated as the number of orders fulfilled divided by number of orders placed, multiplied by hundred.

Order lead times for delivery of goods, which is the number of days that elapsed from ordering to delivery of goods, were extracted from dates of email-based orders and those of delivery notes signed by recipient. Furthermore, data on timely payment (i.e. number of days taken by the health facility to pay Jazia PVS according to contract) were derived from dates of signed quarterly delivery notes and dates of signed payment vouchers to Jazia PVS. Lastly, the satisfaction of pharmacists incharge with performance of Jazia PVS was assessed using a self-administered questionnaire. Questionnaires were pre-tested at two dispensaries not included in the sample, following which adjustments were made to the questions to improve clarity.

### Data processing study variables and analysis

Data were entered in an excel sheet and then exported for analysis to IBM SPSS Statistics for Windows version 25.0 (IBM Corp, Armonk, NY, USA). In this study, there were four outcome variables assessed before and after introduction of Jazia PVS and expressed as percentages. Comparison of availability of health commodities was done using paired *t*-test, at significance level of *P* < 0.05. Order fill rate, order lead time, and timely payment and satisfaction with Jazia PVS among the pharmacists incharge were analysed by using Chi-square test, at a significance level of *P* < 0.05.

## Results

The study included 138 orders made per quarter (every 3 months), totaling 552 orders for 2017 and the same number for 2019. The number of pharmacists incharge interviewed was 138.

### Availability of health commodities before (2017) and after (2019) Jazia PVS commencement

The mean availability of health commodities before and after the introduction of Jazia PVS was 54.39% and 59.17%, respectively, showing a mean increase of 4.78% (Table 1).

**Table 1:** Availability of health commodities before (2017) Jazia PVS and after (2019) Jazia PVS commenced

Period		Availability of health commodities				
Quarter	Year	Minimum (%)	Maximum (%)	Mean (%)	SD (%)	Variance (%)
Jan–March	2017	34	68	51.99	6.629	43.939
	2019	33	75	54	9	80
April–June	2017	34	70	54.01	6.374	40.628
	2019	29	75	58	8	62
July–Sept	2017	39	71	54.90	6.833	46.690
	2019	38	79	61	8	71
Oct–Nov	2017	40	81	56.06	6.893	47.515
	2019	41	85	64	9	76
Average before Jazia PVS^a^	2017	39	70	54.39	5.363	28.765
Average after Jazia PVS	2019	37	74	59.17	6	37
Availability mean increase				4.78		

*SD* standard deviation

^a^*PVS* prime vendor system

Paired *t*-test was used to compare the availability of health commodities in 2017 and 2019, and it showed that after the introduction of Jazia PVS, the mean availability for 2019 (59.17, SD = 6.12) was significantly higher than that of 2017, (Mean = 54.39; SD = 5.36); *t* = − 9.48; *df* = 136; *p* < 0.001 (Tables 2 and 3).

**Table 2:** Comparison of availability of health commodities before and after Jazia PVS adoption

	Mean availability (%)	*N*	SD (%)	SEM (%)
Before Jazia PVS (2017)	54.39	138	5.363	0.457
After Jazia PVS (2019)	59.17	138	6.117	0.521

*PVS* prime vendor system, *SD* standard deviation, *SEM* standard error of the mean

**Table 3:** Mean difference of availability of commodities before and after Jazia Prime Vendor System commenced

Availability	Paired differences (%)					*t*	*df*	*P* value^a^
	Mean	SD		95% CI				
			SEM	Lower	Upper			
Difference after Jazia PVS	4.779	5.917	0.504	3.783	5.775	− 9.488	136	< 001

*SD* standard deviation, *SEM* standard error of the mean, *CI* confidence interval of the difference, *t* *t*-test, *df* degree of freedom

^a^*P* value at 5% level of significance

### Jazia PVS order fill rate

As for the order fill rate, the duly recorded orders were 421 and only these were used in this study. The results showed that 109 (20.3%) orders were fulfilled accordingly, while 312 (58%) were not, but the difference in order fill rates was not statistically significant, Table 4.

**Table 4:** Orders of items fulfilled by Jazia PVS as per requests (*N* = 421)

District Council	Orders fulfilled as per request		Total *n* (%)	Chi-square	*df*	*P* value^a^
	Yes *n* (%)	No *n* (%)				
Ikungi DC	21 (26.3)	59 (73.7)	80 (19.0)	10.46	6	0.1067
Iramba DC	18 (21.2)	67 (78.8)	85 (20.2)			
Itigi DC	16 (33.3)	32 (66.7)	48 (11.4)			
Manyoni DC	11 (18.0)	50 (82.0)	61 (11.9)			
Mkalama DC	12 (23.5)	39 (76.5)	51 (12.1)			
Singida DC	29 (36.3)	51 (63.8)	80 19.0)			
Singida MC	2 (12.5)	14 (87.5)	16 (3.8)			
Total	109 (25.9)	312 (74.1)	421 (100)			

*DC* District Council, *MC* Municipal Council

^a^*P* value at 5% level of significance

### Order lead time for delivery of health commodities by Jazia PVS

A total of 434 delivery notes had been appropriately recorded and were therefore used in this study. The contractual order lead time for delivery of the health commodities to health facilities was less than 14 days. The results showed that, significantly more orders (320, 73.7%) were delivered within the contractual period, than the 114 (26.3%) delivered later, (*χ*^2^ = 121.8, *df* = 6, *P* < 0.001), Table 5.

**Table 5:** Order lead times of delivery of health commodities to health facilities (*N* = 434)

Council	Health commodities delivered on time (less than 14 days)		Total *n* (%)	Chi-square	*df*	*P* value^a^
	Yes *n* (%)	No *n* (%)				
Ikungi DC	68 (72.3)	26 (27.7)	94 (21.7)	121.8	6	< 0.001
Iramba DC	39 (45.9)	46 (54.1)	85 (19.6)			
Itigi DC	18 (37.5)	30 (62.5)	48 (11.1)			
Manyoni DC	61 (100)	0 (0.0)	61 (14.1)			
Mkalama DC	51 (100)	0 (0.0)	51 (11.8)			
Singida DC	73 (92.4)	6 (7.6)	79 (18.2)			
Singida MC	10 (62.5)	6 (37.5)	16 (3.7)			
Total	320 (73.7)	114 (26.3)	434 (100)			

*DC* District Council, *MC* Municipal Council, *df* degree of freedom

^a^*P* value at 5% level of significance

### Timely payment of Jazia Prime Vendor System by health facilities

The 381 payment vouchers that had been duly recorded were used in this study to calculate time taken to pay Jazia PVS. The contractual period for the health facility to pay Jazia PVS was less than 21 days. The results showed that significantly fewer orders (164, 43.0%) were paid within the contractual period by health facilities in different Councils, than the 217 (57.0%) paid later (*χ*^2^ = 26.92, *df* = 6, *p* < 0.001), Table 6.

**Table 6:** Timely payment of Jazia PCS by health facilities for deliveries made (*N* = 381)

Council	Promptness in payment (less than 21 days)		Total *n* (%)	Chi-square	*df*	*P* value^a^
	Prompt *n* (%)	Not prompt *n* (%)				
Ikungi DC	31 (33.0)	63 (67.0)	94 (24.7)	26.92	6	< 0.001
Iramba DC	47 (54.7)	39 (45.3)	86 (22.6)			
Itigi DC	13 (59.1)	9 (40.9)	22 (5.8)			
Manyoni DC	9 (25.0)	27 (75.0)	36 (9.4)			
Mkalama DC	14 (27.5)	37 (72.5)	51 (13.4)			
Singida DC	41 (51.9)	38 (48.1)	79 (20.7)			
Singida MC	9 (69.2)	4 (30.8)	13 (3.4)			
Total	164 (43.0)	217 (57.0)	381 (100)			

*DC* District Council, *MC* Municipal Council, *df* degree of freedom

^a^*P* value at 5% level of significance

### Satisfaction with Jazia PVS by pharmacists incharge

From the results of self-administered questionnaire for pharmacists incharge, 45 (32.61%) indicated that Jazia PVS had improved availability of health commodities, while 93(67.39%) were contrary to that. Regarding Jazia PVS order fill rate, 133 (96.38%) respondents expressed that order fill rate was below 100% and only 5(3.62%) stated that Jazia PVS complied by 100%. Concerning order lead time, 133 (96.38%) pharmacists incharge acknowledged that Jazia PVS order lead time complied with the contract, and only 5 (3.62%) stated the converse. Only 10 (7.25%) of the pharmacists incharge positively recommended Jazia PVS, while 128 (92.75%) did not. Overall, the satisfaction level of the pharmacists incharge with Jazia PVS based on their responses of Yes or No, was 11.8%, (*χ*^2^ = 78.04, *df* = 3, *p* < 0.001), Table 7.

**Table 7:** Level of satisfaction of pharmacists incharge with performance of Jazia Prime Vendor System (*N* = 138)

Questions	Responses		Total	Chi-square	*df*	*P* value^a^
	Yes *n* (%)	No *n* (%)				
i. Has Jazia Prime Vendor improved the availability of the health commodities in your health facility?	45 (32.6)	93 (67.4)	138	78.04	3	< 0.001
ii. Has Jazia Prime Vendor fulfilled the order as per your request?	5 (3.6)	133 (96.4)	138			
iii. Has Jazia Prime Vendor delivered consignment to the district on time?	5 (3.6)	133 (96.4)	138			
iv. Do you recommend Jazia Prime Vendor in complementing Medical Stores Department?	10 (7.2)	128 (92.8)	138			
Total of multiple responses	65 (11.8)^b^	487 (88.2)	552			

*df* degree of freedom

^a^*P* value at 5% level of significance

^b^Overall satisfaction level

## Discussion

The purpose of this study was to assess the performance of Jazia PVS in complementing MSD to deliver health commodities so as to improve their availability in Singida Region, Tanzania. This was achieved by comparing the performance indicators before the adoption of Jazia PVS in 2017 and after in 2019.

### Availability of health commodities before PVS (2017) and after PVS (2019)

The results show that after introducing Jazia PVS, availability of health commodities increased by 4.78% from 54.39% in 2017 to 59.17% in 2019. These results are inconsistent with the study done in Dodoma Region in 2018 by Wiedenmayer et al. [4], which showed that the availability of health commodities in health facilities in the Region increased from 69 to 94% after the introduction of Jazia PVS. The observed difference might be attributed a number possible reasons. First, the Dodoma Region study [4] was conducted after a number of years of piloting Jazia PVS in the area, which might have given the health facility staff and the contracted Jazia PVS considerable experience, and hence better performance compared to the team in Singida Region study [15, 18]. Secondly, it is likely that acceptability of a private vendor (Jazia PVS) was still low, which might have resulted in delays of making orders and payments, hence, low performance of Jazia PVS [19]. The level of health commodity availability in our study was still lower than 80% recommended by WHO [10]. This can be attributed to the fact that the increase in availability of health commodities appears to be incremental, improving gradually with training and experience of the actors [15]. Furthermore, increase in availability of health commodities demands adequacy of budget, high level of transparency, accountability, good governance and management, committed and adequate staff and supervision, as well as reputation of Jazia PVS, which may not be homogeneous across the Regions [13, 16, 20].

### Jazia PVS order fill rate

The results from the study show that Jazia PVS did not satisfy the requests of the health facilities as only 109 (25.9%) of the total orders were fulfilled while 312 (74.1%) orders were not honoured. The order fill rate by Jazia PVS observed in this study is far below the 99% reported in the study conducted in Dodoma Region, Tanzania in 2018 [15]. The discrepancy in these findings might be attributed to Jazia PVS having limited capacity to stock sufficient number of items, to restock the delivered items; financial difficulties due to delayed payments [13, 16]; and by both Jazia PVS and health facilities not to make the right forecasting [21]. Unfulfilled orders could lead to stock-outs at health facilities thereby eroding away the trust of the people, who then resort to out-of-pocket purchase of medicines and other health commodities [22]. To attain universal health coverage, it is essential that out-of-pocket payments by patients for medicines and other health commodities be reduced substantially, by markedly improving order fulfilments [23].

### Order lead time for delivery of health commodities by Jazia PVS

The results of this study have shown that Jazia PVS met the less than 14 days contractual order lead time for delivery of health commodities to health facilities by 73.7% (320), while 26.3% (114) delayed. This is consistent with the results of the studies conducted in Dodoma Region and Dar-es-Salaam in which the lead times conformed to the target of less than 14 days [12, 15]. The performance of the supplier to deliver the consignment on time is influenced by several factors such as distance from the source to the user, reliable transport system from the supplier as well as timely payment by the user to enhance the financial capacity of the supplier to restock the commodities [16]. Shortening lead time for delivery of health commodities reduces stock-outs, the need for overstocking and attendant high holding cost, and expirations; and eventually there is cost saving [24]. Furthermore, short lead time ensures supply chain agility, by being able to manage unpredictable exigencies without causing a shortage. It is therefore essential that short lead times should be taken into account in the selection of suppliers [24].

### Timely payment of Jazia PVS by health facilities

The results have shown that most (217, 57%) health facilities did not pay Jazia PVS on time. This is in accord with earlier study conducted in Dodoma Region to assess the performance of the pilot programme of Jazia PVS which revealed that there was a delay in payment of the supplier by most public health facilities [15]. This is not surprising because delayed payment to suppliers is a universal phenomenon; and it constitutes one of the major challenges in supply chain management across different socio-economic sectors [25, 26]. In health supply chain, delayed payment is mainly attributed to financial constraints and inefficient payment systems on the part of the health facility; and improperly prepared claims by the supplier [14, 27]. Delayed payments can reduce the financial capacity and performance of the supplier thereby increasing the cost, resulting in disruptions down the supply chain and stock-outs [14, 28]. Furthermore, health providers become demoralized that they cannot provide desired service due to stock-out; or ask for informal fee for the few health commodities available [29]. With increased demand for high level of efficiency to ensure availability of heath commodities, there is need for health facilities to enhance the financial position of Jazia PVS by prompt payments after delivery of orders [8]. In addition, both the supplier and the health facilities should strive to utilize finance technology in processing payments for increased efficiency [28].

### Level of satisfaction of pharmacists incharge with performance of Jazia Prime Vendor System

Overall, the satisfaction level of the pharmacists incharge with Jazia PVS based on their responses of “Yes” or “No”, was 11.8%. This finding is in contrast with that of a study conducted in the USA which reported that there was increased satisfaction of vendor system users [30]. There are two likely causes of the differences between the two studies. First, in the USA study there were more than one prime vendor, which could have generated a competitive spirit. Secondly, the USA setting in which the study was conducted might have been more financially and logistically capable than the Singida Region study area. The low level of satisfaction in this study could be attributed to the low availability (54.39%), and low order fill rate (25.9%), which most likely adversely affected the performance of the pharmacists incharge considerably, and hence only 7.2% recommended Jazia PVS in complementing the Medical Stores Department in supply of health commodities [19]. In addition, stock-outs have a tendency to lower the morale of the health care providers and creating among them a negative attitude towards the suppliers.

### Limitations

This study has some imitations that need to be taken into consideration. First, the study used data of one year (i.e. 2019) after the introduction of Jazia PVS due to the effect of COVID-19 on research activities. Therefore, further research is needed to cover a longer period of operation of Jazia PVS. Secondly, some data were missing in the archives, which calls for improvements to be made in record keeping. Thirdly, this study was done in only one Region, and therefore it should be interpreted with caution due to limited generalizability. There is a likelihood of the positive influence of other initiatives in the Region that might have contributed to the results but were not controlled for in the analysis. However, the results from this study can be used to design ways for improvement of Jazia PVS operations, and conducting further studies covering a wider range of Regions, indicators and respondents.

## Conclusion

The percentage availability of the health commodities was found to be 59.17%, which is an increase of 4.78% to pre-evaluation availability two years earlier, just like other studies found over similar time interval, indicating a significant improvement in availability. However, since it is still below the 80% WHO target, more efforts should be made to increase availability of health commodities. The order fill rate was at 25% indicating that many orders were not fulfilled possibly mainly due to delayed payments to Jazia PVS. Jazia PVS met the required order lead time by 73.7% of the time, which is substantial, although there is still a need for improvement to avoid stock-outs. There was a significant delay (57%) in payment of Jazia PVS by the health facilities which might be contributing to low fill rate. Health facilities should ensure Jazia PVS is paid on time possibly by improving financial performance, payment systems and training of health facility staff and by sharing of information. To enhance acceptability of private suppliers, it is essential to create opportunities for sharing information and increased interaction. Further research on this subject should be conducted in more Regions in the country to explore the level of performance of Jazia PVS and associated factors in order to recommend ways for improvement.

## Data Availability

The datasets used during the current study are available from the corresponding author on reasonable request.

## Abbreviations

EAC RCE–VIHSCM: EAC Regional Centre of Excellence for Vaccines, Immunization, and Health Supply Chain Management
GoT: Government of Tanzania
Jazia PVS: Jazia Prime Vendor System
MSD: Medical Stores Department
SOS: Strategic outsourcing system
WHO: World Health Organization
